# (*E*)-1-Ferrocenyl-3-(4-methoxy­phen­yl)­prop-2-en-1-one

**DOI:** 10.1107/S1600536808025518

**Published:** 2008-08-13

**Authors:** Shi-Jia Long, Xiao-Lan Liu, Yong-Hong Liu

**Affiliations:** a College of Life Sciences and Chemistry, Tianshui Normal University, Tianshui 741000, People’s Republic of China; b College of Chemistry and Chemical Engineering, Yangzhou University, Yangzhou 225002, People’s Republic of China

## Abstract

In the title compound, [Fe(C_5_H_5_)(C_15_H_13_O_2_)], a conjugated substituent group bridges a five-membered η^5^-C_5_H_4_ ring and a benzene ring. In the ferrocene unit, the substituted (Cps) and unsubstituted (Cp) cyclo­penta­dienyl rings are eclipsed and almost parallel [Cps—Fe—Cps angle = 176.1 (2)°]. The mol­ecule is linked into an *S*(5) motif *via* intra­molecular C—H⋯O hydrogen bonds. The mol­ecules are arranged into a three-dimensional framework by five inter­molecular C—H⋯O hydrogen bonds and one inter­molecular C—H⋯π(Cps) inter­action.

## Related literature

For related literature, see: Bernstein *et al.* (1995 ▶); Edwards *et al.* (1975 ▶); Huang *et al.* (1998 ▶); Liang *et al.* (1998 ▶); Liu *et al.* (2001 ▶, 2003 ▶, 2008 ▶); Shi *et al.* (2004 ▶); Yarishkin *et al.* (2008 ▶); Zhai *et al.* (1999 ▶).
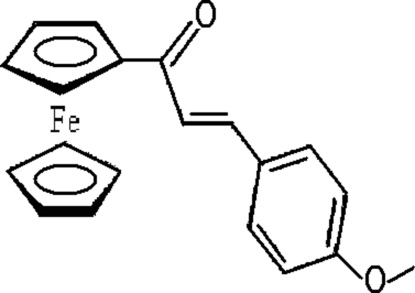


## Experimental

### 

#### Crystal data

[Fe(C_5_H_5_)(C_15_H_13_O_2_)]
*M* *_r_* = 346.19
Orthorhombic, 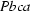

*a* = 12.3124 (14) Å
*b* = 10.2316 (11) Å
*c* = 25.914 (3) Å
*V* = 3264.5 (6) Å^3^
*Z* = 8
Mo *K*α radiation
μ = 0.93 mm^−1^
*T* = 296 (2) K
0.30 × 0.30 × 0.20 mm

#### Data collection

Bruker SMART 1000 CCD diffractometer
Absorption correction: multi-scan (*SADABS*; Bruker, 2002 ▶) *T*_min_ = 0.768, *T*_max_ = 0.836
26999 measured reflections
3787 independent reflections
2662 reflections with *I* > 2σ(*I*)
*R*_int_ = 0.037

#### Refinement

*R*[*F* ^2^ > 2σ(*F* ^2^)] = 0.032
*wR*(*F* ^2^) = 0.089
*S* = 1.02
3787 reflections
209 parameters
H-atom parameters constrained
Δρ_max_ = 0.20 e Å^−3^
Δρ_min_ = −0.28 e Å^−3^

### 

Data collection: *SMART* (Bruker, 2002 ▶); cell refinement: *SAINT* (Bruker, 2002 ▶); data reduction: *SAINT*; program(s) used to solve structure: *SHELXS97* (Sheldrick, 2008 ▶); program(s) used to refine structure: *SHELXL97* (Sheldrick, 2008 ▶); molecular graphics: *PLATON* (Spek, 2003 ▶); software used to prepare material for publication: *SHELXTL* (Sheldrick, 2008 ▶).

## Supplementary Material

Crystal structure: contains datablocks I, global. DOI: 10.1107/S1600536808025518/pv2096sup1.cif

Structure factors: contains datablocks I. DOI: 10.1107/S1600536808025518/pv2096Isup2.hkl

Additional supplementary materials:  crystallographic information; 3D view; checkCIF report

## Figures and Tables

**Table 1:** Hydrogen-bond geometry (Å, °) *Cg*1 is the centroid of the substituted cyclo­penta­dienyl ring.

*D*—H⋯*A*	*D*—H	H⋯*A*	*D*⋯*A*	*D*—H⋯*A*
C13—H13⋯O1	0.93	2.50	2.830 (2)	101
C8—H8⋯O1^i^	0.93	2.60	3.345 (2)	137
C9—H9⋯O1^ii^	0.93	2.54	3.414 (2)	156
C12—H12⋯O1^ii^	0.93	2.46	3.346 (2)	160
C15—H15⋯O1^ii^	0.93	2.65	3.441 (2)	143
C19—H19⋯O2^iii^	0.93	2.59	3.472 (3)	157
C3—H3⋯*Cg*1^i^	0.93	3.24	3.808 (2)	121

Symmetry codes: (i) 
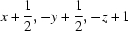
; (ii) 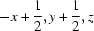; (iii) 
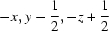
.

**Fig. 1.**
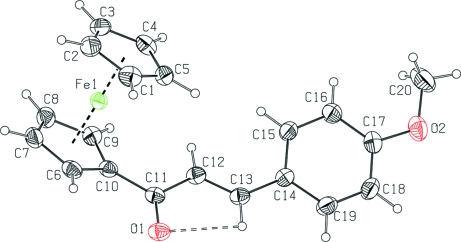
The molecular structure of the title compound, showing 30% probability displacement ellipsoids. The C–H···O intra-molecular hydrogen bond is shown as dashed lines.

**Fig. 2.**
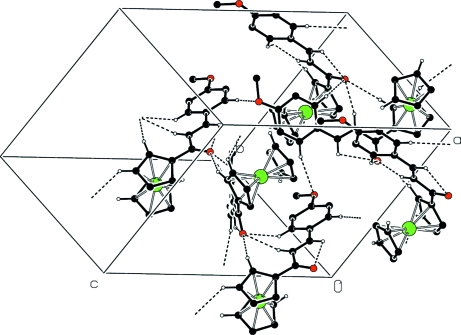
Unit cell packing of (I) showing the hydrogen bonded chains (dashed lines). For clarity, H atoms not involved in hydrogen bonding have been omitted.

**Fig. 3.**
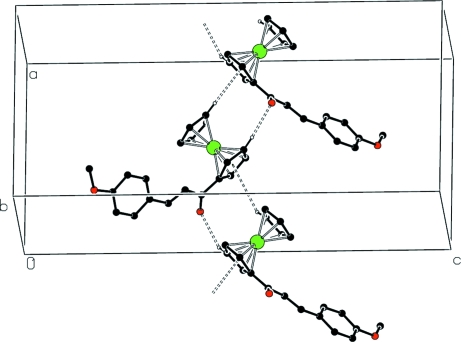
Unit cell packing of (I) showing the hydrogen bonded chains via C8–H8···O1 and C3–H3···π(Cps ring) inter-molecular interactions; for clarity, H atoms not involved in hydrogen bonding have been omitted.

**Fig. 4.**
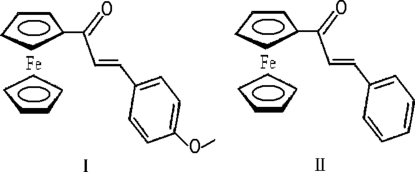
Schematic representations of the structures of (I) and (II).

## supplementary crystallographic information

### Comment

Chalcone and its derivatives, as natural products, have attracted considerable attention for their stronge antibacterial, antifungal, antitumor and anti-inflammatory properties, especially antileishmanial and antimalarial (Zhai *et al.*,1999; Liu *et al.*, 2001, 2003) over the past years. Some chalcones demonstrated the ability to block voltage-dependent potassium channels (Yarishkin *et al.*, 2008). It was proved that the replacement of the aromatic group by the ferrocenyl moiety in penicillins and cephalosporins improves their antibiotic activity (Edwards *et al.*, 1975). As part of our search for new biologically active compounds (Liu *et al.*,2008; Shi *et al.*, 2004; Liang *et al.*, 1998), we have synthesized the title compound (I) and describe its structure in this paper.

The molecule of the title compound (I) exists as the most stable configuration of (*E)*-isomer (Scheme 1, Fig.1) and all carbon atoms (except that of methoxyl group) and O1 are *sp*^2^-hybridized and form two large conjugated systems; one is formed by C1 to C5 and the other C6 to C19 including O1, just as its parent compound II (Scheme 2) (Liu *et al.*, 2008). In the molecule there is a *C(5)* (Bernstein *et al.*, 1995) C–H···O intra-molecular hydrogen-bond which makes the four atoms O1, C11, C12 and C13 be coplanar (plane-1). The Cps (the substituted cyclopentadienyl ring) plane, the plane-1 and plane-2 (the phenyl ring plane) are not coplanar. In the ferrocene moiety, the Cps and the Cp (the unsubstituted cyclopentadienyl ring) planes are almost parallel and the C-atoms of these tings are in an eclipsed conformation. The Fe atom lies in the middle of the two planes of Cp and Cps. The Cgs—Fe—*Cg* angle is 176.1 (2)°, where Cgs and *Cg* are the centroids of Cps and Cp rings, respectively.

The molecules are linked into *C(5)* (Bernstein *et al.*, 1995) chains *via* C19–H19···O1 inter-molecular hydrogen-bonds, forming zigzag chains (Fig. 2, Table 1) along the *b* axis. In addition, there are three inter-molecular hydrogen-bonds C9–H9···O1, C12–H12···O1 and C15–H15···O1, thus forming cross edge-fused zigzag *C(5), C(4), C(7)* chains (Fig. 2) along the *b* axis. Furthermore, the molecules are linked into *C(6)* (Bernstein *et al.*, 1995) chains *via* C8–H8···O1 and C3–H3···*π*(Cps ring) inter-molecular hydrogen-bonds, along the *a* axis thus resulting in other zigzag chains (Fig. 3, Table 1). All of the above mentioned inter-molecular hydrogen-bonds link the molecules into a three-dimensional structure of considerable complexity.

### Experimental

The title compound was synthesized according to the literature procedure (Huang *et al.* 1998). Crystals of I suitable for X-ray diffraction were obtained by slow evaporation of a solution of the solid in dichloromethane / ether (5:1 *v*/*v*) at room temperature over a period of 6 days.

### Refinement

After their location in a difference map, all H atoms were fixed geometrically at ideal positions and allowed to ride on the parent C atoms, with C—H distances of 0.93 – 0.96, and with U_ĩso_(H) values of *1.2U*_eq_ (C).

### Crystal data

**Table:**

[Fe(C_5_H_5_)(C_15_H_13_O_2_)]	*F*_000_ = 1440
*M**_r_* = 346.19	*D*_x_ = 1.409 Mg m^−^^3^
Orthorhombic, *P**b**c**a*	Mo *K*α radiation λ = 0.71073 Å
Hall symbol: -P 2ac 2ab	Cell parameters from 6158 reflections
*a* = 12.3124 (14) Å	θ = 3.0–25.5º
*b* = 10.2316 (11) Å	µ = 0.93 mm^−^^1^
*c* = 25.914 (3) Å	*T* = 296 (2) K
*V* = 3264.5 (6) Å^3^	Block, red
*Z* = 8	0.30 × 0.30 × 0.20 mm

### Data collection

**Table:**

Bruker SMART 1000 CCD diffractometer	3787 independent reflections
Radiation source: fine-focus sealed tube	2662 reflections with *I* > 2σ(*I*)
Monochromator: graphite	*R*_int_ = 0.037
*T* = 296(2) K	θ_max_ = 27.6º
φ and ω scans	θ_min_ = 1.6º
Absorption correction: multi-scan(SADABS; Bruker, 2002)	*h* = −14→16
*T*_min_ = 0.768, *T*_max_ = 0.836	*k* = −13→13
26999 measured reflections	*l* = −33→33

### Refinement

**Table:**

Refinement on *F*^2^	Secondary atom site location: difference Fourier map
Least-squares matrix: full	Hydrogen site location: inferred from neighbouring sites
*R*[*F*^2^ > 2σ(*F*^2^)] = 0.032	H-atom parameters constrained
*wR*(*F*^2^) = 0.089	*w* = 1/[σ^2^(*F*_o_^2^) + (0.0396*P*)^2^ + 0.9963*P*] where *P* = (*F*_o_^2^ + 2*F*_c_^2^)/3
*S* = 1.02	(Δ/σ)_max_ = 0.001
3787 reflections	Δρ_max_ = 0.20 e Å^−^^3^
209 parameters	Δρ_min_ = −0.28 e Å^−^^3^
Primary atom site location: structure-invariant direct methods	Extinction correction: none

### Special details

**Table:**

Geometry. All e.s.d.'s (except the e.s.d. in the dihedral angle between two l.s. planes) are estimated using the full covariance matrix. The cell e.s.d.'s are taken into account individually in the estimation of e.s.d.'s in distances, angles and torsion angles; correlations between e.s.d.'s in cell parameters are only used when they are defined by crystal symmetry. An approximate (isotropic) treatment of cell e.s.d.'s is used for estimating e.s.d.'s involving l.s. planes.
Refinement. Refinement of *F*^2^ against ALL reflections. The weighted *R*-factor *wR* and goodness of fit *S* are based on *F*^2^, conventional *R*-factors *R* are based on *F*, with *F* set to zero for negative *F*^2^. The threshold expression of *F*^2^ > σ(*F*^2^) is used only for calculating *R*-factors(gt) *etc*. and is not relevant to the choice of reflections for refinement. *R*-factors based on *F*^2^ are statistically about twice as large as those based on *F*, and *R*- factors based on ALL data will be even larger.

### Fractional atomic coordinates and isotropic or equivalent isotropic displacement parameters (Å^2^)

**Table:**

	*x*	*y*	*z*	*U*_iso_*/*U*_eq_	
Fe1	0.47330 (2)	0.24558 (2)	0.447598 (9)	0.03889 (10)	
O1	0.17057 (11)	0.14263 (12)	0.41478 (5)	0.0509 (3)	
C14	0.14386 (15)	0.45292 (18)	0.30462 (7)	0.0423 (4)	
C12	0.22120 (15)	0.35247 (17)	0.38469 (7)	0.0424 (4)	
H12	0.2671	0.4235	0.3898	0.051*	
C17	0.12977 (17)	0.6313 (2)	0.22287 (7)	0.0502 (5)	
C15	0.22061 (16)	0.5508 (2)	0.29796 (7)	0.0502 (5)	
H15	0.2777	0.5574	0.3213	0.060*	
C13	0.15074 (15)	0.35526 (18)	0.34563 (7)	0.0433 (4)	
H13	0.1004	0.2876	0.3444	0.052*	
C16	0.21475 (17)	0.6392 (2)	0.25751 (7)	0.0530 (5)	
H16	0.2677	0.7033	0.2537	0.064*	
C19	0.05897 (17)	0.4478 (2)	0.26898 (8)	0.0552 (5)	
H19	0.0062	0.3833	0.2723	0.066*	
C11	0.22818 (15)	0.23985 (17)	0.41997 (7)	0.0405 (4)	
C18	0.05177 (18)	0.5362 (2)	0.22907 (8)	0.0589 (6)	
H18	−0.0062	0.5316	0.2061	0.071*	
C7	0.43869 (17)	0.1790 (2)	0.52041 (8)	0.0553 (5)	
H7	0.4829	0.1267	0.5409	0.066*	
C9	0.36118 (15)	0.35924 (19)	0.48277 (7)	0.0454 (4)	
H9	0.3459	0.4455	0.4740	0.055*	
C1	0.54687 (18)	0.1382 (2)	0.39076 (8)	0.0580 (5)	
H1	0.5308	0.0523	0.3818	0.070*	
C6	0.36007 (15)	0.13343 (19)	0.48474 (8)	0.0495 (5)	
H6	0.3436	0.0465	0.4778	0.059*	
C10	0.31062 (15)	0.24527 (17)	0.46128 (7)	0.0418 (4)	
C5	0.49898 (18)	0.2505 (2)	0.36970 (8)	0.0548 (5)	
H5	0.4455	0.2521	0.3443	0.066*	
C2	0.62385 (17)	0.1786 (2)	0.42794 (8)	0.0600 (6)	
H2	0.6674	0.1240	0.4478	0.072*	
C3	0.62294 (17)	0.3156 (2)	0.42965 (8)	0.0582 (6)	
H3	0.6658	0.3677	0.4509	0.070*	
C8	0.43881 (17)	0.3170 (2)	0.51975 (7)	0.0529 (5)	
H8	0.4822	0.3709	0.5400	0.064*	
C4	0.54580 (18)	0.3608 (2)	0.39360 (8)	0.0569 (5)	
H4	0.5288	0.4477	0.3868	0.068*	
O2	0.11598 (14)	0.71299 (17)	0.18152 (6)	0.0679 (4)	
C20	0.1979 (2)	0.8096 (3)	0.17227 (11)	0.0906 (9)	
H20A	0.1996	0.8703	0.2005	0.136*	
H20B	0.1818	0.8555	0.1409	0.136*	
H20C	0.2674	0.7677	0.1692	0.136*	

### Atomic displacement parameters (Å^2^)

**Table:**

	*U*^11^	*U*^22^	*U*^33^	*U*^12^	*U*^13^	*U*^23^
Fe1	0.03704 (16)	0.03933 (16)	0.04030 (15)	0.00167 (11)	0.00085 (10)	0.00384 (11)
O1	0.0463 (7)	0.0378 (7)	0.0686 (8)	−0.0054 (6)	0.0019 (7)	0.0049 (6)
C14	0.0404 (10)	0.0407 (10)	0.0458 (10)	0.0023 (8)	−0.0012 (8)	−0.0031 (8)
C12	0.0399 (10)	0.0333 (9)	0.0541 (10)	0.0000 (8)	−0.0016 (8)	0.0020 (8)
C17	0.0562 (12)	0.0520 (12)	0.0425 (10)	0.0149 (10)	0.0028 (9)	0.0008 (9)
C15	0.0477 (11)	0.0524 (12)	0.0506 (11)	−0.0053 (10)	−0.0095 (9)	0.0036 (9)
C13	0.0391 (10)	0.0364 (10)	0.0545 (10)	−0.0007 (8)	0.0011 (8)	−0.0001 (8)
C16	0.0561 (13)	0.0490 (12)	0.0541 (11)	−0.0040 (10)	−0.0012 (10)	0.0052 (9)
C19	0.0453 (11)	0.0578 (13)	0.0626 (13)	−0.0075 (10)	−0.0082 (10)	−0.0002 (10)
C11	0.0362 (9)	0.0348 (9)	0.0504 (10)	0.0040 (8)	0.0076 (7)	0.0014 (8)
C18	0.0518 (12)	0.0698 (15)	0.0552 (12)	0.0036 (11)	−0.0159 (10)	0.0025 (11)
C7	0.0519 (12)	0.0666 (14)	0.0475 (11)	0.0084 (11)	0.0023 (10)	0.0179 (10)
C9	0.0462 (10)	0.0434 (10)	0.0466 (10)	0.0056 (9)	0.0033 (9)	−0.0014 (8)
C1	0.0591 (13)	0.0538 (13)	0.0613 (13)	0.0016 (11)	0.0120 (11)	−0.0105 (11)
C6	0.0465 (11)	0.0434 (11)	0.0586 (11)	0.0025 (9)	0.0083 (10)	0.0162 (9)
C10	0.0360 (9)	0.0413 (10)	0.0481 (9)	0.0032 (8)	0.0064 (8)	0.0073 (8)
C5	0.0491 (11)	0.0756 (16)	0.0397 (10)	−0.0032 (11)	0.0015 (8)	0.0007 (10)
C2	0.0431 (12)	0.0792 (17)	0.0578 (12)	0.0134 (11)	0.0053 (10)	0.0048 (12)
C3	0.0454 (12)	0.0774 (16)	0.0516 (12)	−0.0177 (11)	0.0040 (10)	−0.0041 (11)
C8	0.0526 (12)	0.0649 (14)	0.0412 (10)	0.0058 (10)	0.0021 (9)	−0.0020 (10)
C4	0.0665 (14)	0.0525 (13)	0.0518 (11)	−0.0069 (11)	0.0112 (10)	0.0099 (10)
O2	0.0735 (11)	0.0751 (10)	0.0552 (8)	0.0178 (9)	0.0000 (8)	0.0171 (8)
C20	0.0817 (19)	0.103 (2)	0.0877 (18)	0.0128 (17)	0.0247 (16)	0.0454 (17)

### Geometric parameters (Å, °)

**Table:**

Fe1—C9	2.0220 (18)	C11—C10	1.476 (3)
Fe1—C3	2.031 (2)	C18—H18	0.9300
Fe1—C10	2.0341 (18)	C7—C8	1.412 (3)
Fe1—C4	2.036 (2)	C7—C6	1.417 (3)
Fe1—C2	2.041 (2)	C7—H7	0.9300
Fe1—C5	2.044 (2)	C9—C8	1.421 (3)
Fe1—C6	2.0462 (18)	C9—C10	1.434 (3)
Fe1—C1	2.049 (2)	C9—H9	0.9300
Fe1—C7	2.0507 (19)	C1—C5	1.403 (3)
Fe1—C8	2.052 (2)	C1—C2	1.414 (3)
O1—C11	1.229 (2)	C1—H1	0.9300
C14—C15	1.388 (3)	C6—C10	1.432 (2)
C14—C19	1.396 (3)	C6—H6	0.9300
C14—C13	1.461 (2)	C5—C4	1.410 (3)
C12—C13	1.333 (2)	C5—H5	0.9300
C12—C11	1.473 (2)	C2—C3	1.403 (3)
C12—H12	0.9300	C2—H2	0.9300
C17—O2	1.369 (2)	C3—C4	1.410 (3)
C17—C18	1.377 (3)	C3—H3	0.9300
C17—C16	1.381 (3)	C8—H8	0.9300
C15—C16	1.386 (3)	C4—H4	0.9300
C15—H15	0.9300	O2—C20	1.433 (3)
C13—H13	0.9300	C20—H20A	0.9600
C16—H16	0.9300	C20—H20B	0.9600
C19—C18	1.377 (3)	C20—H20C	0.9600
C19—H19	0.9300		
			
C9—Fe1—C3	121.31 (9)	C19—C18—H18	119.8
C9—Fe1—C10	41.42 (7)	C17—C18—H18	119.8
C3—Fe1—C10	159.03 (9)	C8—C7—C6	108.78 (18)
C9—Fe1—C4	106.03 (9)	C8—C7—Fe1	69.92 (11)
C3—Fe1—C4	40.57 (9)	C6—C7—Fe1	69.59 (11)
C10—Fe1—C4	123.54 (8)	C8—C7—H7	125.6
C9—Fe1—C2	157.77 (9)	C6—C7—H7	125.6
C3—Fe1—C2	40.30 (10)	Fe1—C7—H7	126.5
C10—Fe1—C2	159.61 (9)	C8—C9—C10	107.85 (17)
C4—Fe1—C2	67.95 (9)	C8—C9—Fe1	70.72 (11)
C9—Fe1—C5	122.45 (8)	C10—C9—Fe1	69.74 (10)
C3—Fe1—C5	67.96 (9)	C8—C9—H9	126.1
C10—Fe1—C5	108.94 (8)	C10—C9—H9	126.1
C4—Fe1—C5	40.44 (8)	Fe1—C9—H9	125.1
C2—Fe1—C5	67.74 (9)	C5—C1—C2	107.9 (2)
C9—Fe1—C6	69.23 (8)	C5—C1—Fe1	69.76 (12)
C3—Fe1—C6	157.48 (9)	C2—C1—Fe1	69.47 (12)
C10—Fe1—C6	41.08 (7)	C5—C1—H1	126.1
C4—Fe1—C6	161.23 (9)	C2—C1—H1	126.1
C2—Fe1—C6	123.23 (9)	Fe1—C1—H1	126.3
C5—Fe1—C6	125.73 (9)	C7—C6—C10	107.71 (18)
C9—Fe1—C1	159.13 (9)	C7—C6—Fe1	69.93 (11)
C3—Fe1—C1	67.89 (9)	C10—C6—Fe1	69.00 (10)
C10—Fe1—C1	124.04 (8)	C7—C6—H6	126.1
C4—Fe1—C1	67.83 (10)	C10—C6—H6	126.1
C2—Fe1—C1	40.44 (9)	Fe1—C6—H6	126.5
C5—Fe1—C1	40.09 (8)	C6—C10—C9	107.48 (17)
C6—Fe1—C1	109.79 (9)	C6—C10—C11	124.77 (17)
C9—Fe1—C7	68.56 (8)	C9—C10—C11	127.62 (16)
C3—Fe1—C7	121.09 (9)	C6—C10—Fe1	69.91 (10)
C10—Fe1—C7	68.56 (8)	C9—C10—Fe1	68.84 (10)
C4—Fe1—C7	156.32 (9)	C11—C10—Fe1	123.41 (13)
C2—Fe1—C7	107.87 (9)	C1—C5—C4	108.2 (2)
C5—Fe1—C7	161.64 (9)	C1—C5—Fe1	70.15 (12)
C6—Fe1—C7	40.48 (8)	C4—C5—Fe1	69.47 (11)
C1—Fe1—C7	125.11 (9)	C1—C5—H5	125.9
C9—Fe1—C8	40.82 (8)	C4—C5—H5	125.9
C3—Fe1—C8	105.71 (9)	Fe1—C5—H5	126.1
C10—Fe1—C8	68.77 (8)	C3—C2—C1	108.00 (19)
C4—Fe1—C8	120.73 (9)	C3—C2—Fe1	69.48 (12)
C2—Fe1—C8	122.34 (9)	C1—C2—Fe1	70.09 (12)
C5—Fe1—C8	157.40 (9)	C3—C2—H2	126.0
C6—Fe1—C8	68.28 (9)	C1—C2—H2	126.0
C1—Fe1—C8	159.65 (9)	Fe1—C2—H2	126.0
C7—Fe1—C8	40.25 (9)	C2—C3—C4	108.18 (19)
C15—C14—C19	117.06 (18)	C2—C3—Fe1	70.23 (12)
C15—C14—C13	123.00 (17)	C4—C3—Fe1	69.89 (12)
C19—C14—C13	119.92 (18)	C2—C3—H3	125.9
C13—C12—C11	121.71 (17)	C4—C3—H3	125.9
C13—C12—H12	119.1	Fe1—C3—H3	125.5
C11—C12—H12	119.1	C7—C8—C9	108.16 (19)
O2—C17—C18	115.87 (19)	C7—C8—Fe1	69.83 (12)
O2—C17—C16	124.5 (2)	C9—C8—Fe1	68.47 (11)
C18—C17—C16	119.58 (18)	C7—C8—H8	125.9
C16—C15—C14	121.96 (18)	C9—C8—H8	125.9
C16—C15—H15	119.0	Fe1—C8—H8	127.4
C14—C15—H15	119.0	C3—C4—C5	107.7 (2)
C12—C13—C14	127.19 (17)	C3—C4—Fe1	69.53 (11)
C12—C13—H13	116.4	C5—C4—Fe1	70.09 (12)
C14—C13—H13	116.4	C3—C4—H4	126.1
C17—C16—C15	119.55 (19)	C5—C4—H4	126.1
C17—C16—H16	120.2	Fe1—C4—H4	125.8
C15—C16—H16	120.2	C17—O2—C20	117.68 (19)
C18—C19—C14	121.4 (2)	O2—C20—H20A	109.5
C18—C19—H19	119.3	O2—C20—H20B	109.5
C14—C19—H19	119.3	H20A—C20—H20B	109.5
O1—C11—C12	122.11 (17)	O2—C20—H20C	109.5
O1—C11—C10	120.43 (16)	H20A—C20—H20C	109.5
C12—C11—C10	117.43 (16)	H20B—C20—H20C	109.5
C19—C18—C17	120.48 (19)		
			
C19—C14—C15—C16	−0.7 (3)	C8—Fe1—C10—C9	38.10 (11)
C13—C14—C15—C16	177.32 (18)	C9—Fe1—C10—C11	121.93 (19)
C11—C12—C13—C14	−172.39 (17)	C3—Fe1—C10—C11	81.7 (3)
C15—C14—C13—C12	7.6 (3)	C4—Fe1—C10—C11	46.44 (18)
C19—C14—C13—C12	−174.41 (19)	C2—Fe1—C10—C11	−72.9 (3)
O2—C17—C16—C15	−179.85 (19)	C5—Fe1—C10—C11	3.99 (17)
C18—C17—C16—C15	0.2 (3)	C6—Fe1—C10—C11	−119.1 (2)
C14—C15—C16—C17	0.7 (3)	C1—Fe1—C10—C11	−37.90 (18)
C15—C14—C19—C18	−0.1 (3)	C7—Fe1—C10—C11	−156.61 (17)
C13—C14—C19—C18	−178.24 (19)	C8—Fe1—C10—C11	160.03 (17)
C13—C12—C11—O1	0.9 (3)	C2—C1—C5—C4	0.0 (2)
C13—C12—C11—C10	178.68 (17)	Fe1—C1—C5—C4	59.23 (15)
C14—C19—C18—C17	1.0 (3)	C2—C1—C5—Fe1	−59.23 (14)
O2—C17—C18—C19	179.00 (19)	C9—Fe1—C5—C1	−164.59 (12)
C16—C17—C18—C19	−1.1 (3)	C3—Fe1—C5—C1	81.40 (14)
C9—Fe1—C7—C8	−37.40 (12)	C10—Fe1—C5—C1	−120.76 (13)
C3—Fe1—C7—C8	77.12 (15)	C4—Fe1—C5—C1	119.4 (2)
C10—Fe1—C7—C8	−82.06 (13)	C2—Fe1—C5—C1	37.73 (13)
C4—Fe1—C7—C8	43.6 (3)	C6—Fe1—C5—C1	−78.05 (15)
C2—Fe1—C7—C8	119.28 (13)	C7—Fe1—C5—C1	−41.9 (3)
C5—Fe1—C7—C8	−167.7 (2)	C8—Fe1—C5—C1	159.1 (2)
C6—Fe1—C7—C8	−120.12 (17)	C9—Fe1—C5—C4	76.03 (15)
C1—Fe1—C7—C8	160.61 (13)	C3—Fe1—C5—C4	−37.98 (14)
C9—Fe1—C7—C6	82.72 (13)	C10—Fe1—C5—C4	119.86 (13)
C3—Fe1—C7—C6	−162.76 (12)	C2—Fe1—C5—C4	−81.64 (15)
C10—Fe1—C7—C6	38.06 (11)	C6—Fe1—C5—C4	162.57 (12)
C4—Fe1—C7—C6	163.7 (2)	C1—Fe1—C5—C4	−119.4 (2)
C2—Fe1—C7—C6	−120.60 (13)	C7—Fe1—C5—C4	−161.3 (2)
C5—Fe1—C7—C6	−47.6 (3)	C8—Fe1—C5—C4	39.8 (3)
C1—Fe1—C7—C6	−79.27 (15)	C5—C1—C2—C3	0.1 (2)
C8—Fe1—C7—C6	120.12 (17)	Fe1—C1—C2—C3	−59.36 (14)
C3—Fe1—C9—C8	−77.33 (14)	C5—C1—C2—Fe1	59.41 (15)
C10—Fe1—C9—C8	118.37 (16)	C9—Fe1—C2—C3	−40.4 (3)
C4—Fe1—C9—C8	−118.72 (13)	C10—Fe1—C2—C3	166.3 (2)
C2—Fe1—C9—C8	−48.0 (3)	C4—Fe1—C2—C3	37.87 (12)
C5—Fe1—C9—C8	−159.64 (13)	C5—Fe1—C2—C3	81.70 (13)
C6—Fe1—C9—C8	80.43 (13)	C6—Fe1—C2—C3	−159.21 (12)
C1—Fe1—C9—C8	171.7 (2)	C1—Fe1—C2—C3	119.11 (18)
C7—Fe1—C9—C8	36.90 (13)	C7—Fe1—C2—C3	−117.30 (13)
C3—Fe1—C9—C10	164.30 (11)	C8—Fe1—C2—C3	−75.45 (15)
C4—Fe1—C9—C10	122.91 (12)	C9—Fe1—C2—C1	−159.5 (2)
C2—Fe1—C9—C10	−166.3 (2)	C3—Fe1—C2—C1	−119.11 (18)
C5—Fe1—C9—C10	81.99 (13)	C10—Fe1—C2—C1	47.2 (3)
C6—Fe1—C9—C10	−37.94 (11)	C4—Fe1—C2—C1	−81.24 (15)
C1—Fe1—C9—C10	53.3 (3)	C5—Fe1—C2—C1	−37.41 (13)
C7—Fe1—C9—C10	−81.47 (12)	C6—Fe1—C2—C1	81.68 (15)
C8—Fe1—C9—C10	−118.37 (16)	C7—Fe1—C2—C1	123.59 (14)
C9—Fe1—C1—C5	39.0 (3)	C8—Fe1—C2—C1	165.44 (13)
C3—Fe1—C1—C5	−81.58 (14)	C1—C2—C3—C4	−0.1 (2)
C10—Fe1—C1—C5	78.79 (15)	Fe1—C2—C3—C4	−59.83 (14)
C4—Fe1—C1—C5	−37.62 (13)	C1—C2—C3—Fe1	59.74 (14)
C2—Fe1—C1—C5	−119.17 (19)	C9—Fe1—C3—C2	163.33 (12)
C6—Fe1—C1—C5	122.43 (13)	C10—Fe1—C3—C2	−166.6 (2)
C7—Fe1—C1—C5	165.10 (13)	C4—Fe1—C3—C2	−118.99 (18)
C8—Fe1—C1—C5	−156.8 (2)	C5—Fe1—C3—C2	−81.13 (13)
C9—Fe1—C1—C2	158.2 (2)	C6—Fe1—C3—C2	50.8 (3)
C3—Fe1—C1—C2	37.58 (14)	C1—Fe1—C3—C2	−37.71 (12)
C10—Fe1—C1—C2	−162.04 (13)	C7—Fe1—C3—C2	80.97 (14)
C4—Fe1—C1—C2	81.55 (15)	C8—Fe1—C3—C2	121.84 (13)
C5—Fe1—C1—C2	119.17 (19)	C9—Fe1—C3—C4	−77.69 (15)
C6—Fe1—C1—C2	−118.41 (14)	C10—Fe1—C3—C4	−47.7 (3)
C7—Fe1—C1—C2	−75.73 (16)	C2—Fe1—C3—C4	118.99 (18)
C8—Fe1—C1—C2	−37.7 (3)	C5—Fe1—C3—C4	37.86 (13)
C8—C7—C6—C10	0.2 (2)	C6—Fe1—C3—C4	169.8 (2)
Fe1—C7—C6—C10	−58.86 (13)	C1—Fe1—C3—C4	81.27 (15)
C8—C7—C6—Fe1	59.10 (14)	C7—Fe1—C3—C4	−160.05 (13)
C9—Fe1—C6—C7	−80.92 (13)	C8—Fe1—C3—C4	−119.17 (13)
C3—Fe1—C6—C7	41.5 (3)	C6—C7—C8—C9	−1.0 (2)
C10—Fe1—C6—C7	−119.15 (17)	Fe1—C7—C8—C9	57.86 (13)
C4—Fe1—C6—C7	−159.5 (2)	C6—C7—C8—Fe1	−58.90 (14)
C2—Fe1—C6—C7	78.32 (15)	C10—C9—C8—C7	1.4 (2)
C5—Fe1—C6—C7	163.36 (13)	Fe1—C9—C8—C7	−58.70 (14)
C1—Fe1—C6—C7	121.33 (13)	C10—C9—C8—Fe1	60.13 (13)
C8—Fe1—C6—C7	−36.99 (12)	C9—Fe1—C8—C7	120.13 (18)
C9—Fe1—C6—C10	38.24 (11)	C3—Fe1—C8—C7	−119.86 (13)
C3—Fe1—C6—C10	160.7 (2)	C10—Fe1—C8—C7	81.49 (13)
C4—Fe1—C6—C10	−40.3 (3)	C4—Fe1—C8—C7	−161.21 (13)
C2—Fe1—C6—C10	−162.52 (12)	C2—Fe1—C8—C7	−79.30 (15)
C5—Fe1—C6—C10	−77.48 (14)	C5—Fe1—C8—C7	169.9 (2)
C1—Fe1—C6—C10	−119.52 (12)	C6—Fe1—C8—C7	37.19 (12)
C7—Fe1—C6—C10	119.15 (17)	C1—Fe1—C8—C7	−51.3 (3)
C8—Fe1—C6—C10	82.17 (12)	C3—Fe1—C8—C9	120.01 (13)
C7—C6—C10—C9	0.6 (2)	C10—Fe1—C8—C9	−38.64 (11)
Fe1—C6—C10—C9	−58.79 (13)	C4—Fe1—C8—C9	78.66 (14)
C7—C6—C10—C11	176.81 (17)	C2—Fe1—C8—C9	160.57 (13)
Fe1—C6—C10—C11	117.38 (18)	C5—Fe1—C8—C9	49.8 (3)
C7—C6—C10—Fe1	59.44 (13)	C6—Fe1—C8—C9	−82.94 (13)
C8—C9—C10—C6	−1.3 (2)	C1—Fe1—C8—C9	−171.5 (2)
Fe1—C9—C10—C6	59.47 (13)	C7—Fe1—C8—C9	−120.13 (18)
C8—C9—C10—C11	−177.31 (17)	C2—C3—C4—C5	0.1 (2)
Fe1—C9—C10—C11	−116.56 (19)	Fe1—C3—C4—C5	−59.95 (14)
C8—C9—C10—Fe1	−60.75 (13)	C2—C3—C4—Fe1	60.04 (14)
O1—C11—C10—C6	24.6 (3)	C1—C5—C4—C3	−0.1 (2)
C12—C11—C10—C6	−153.18 (17)	Fe1—C5—C4—C3	59.60 (14)
O1—C11—C10—C9	−159.99 (18)	C1—C5—C4—Fe1	−59.65 (15)
C12—C11—C10—C9	22.2 (3)	C9—Fe1—C4—C3	119.71 (14)
O1—C11—C10—Fe1	112.17 (18)	C10—Fe1—C4—C3	161.50 (13)
C12—C11—C10—Fe1	−65.6 (2)	C2—Fe1—C4—C3	−37.62 (14)
C9—Fe1—C10—C6	−118.98 (16)	C5—Fe1—C4—C3	−118.72 (19)
C3—Fe1—C10—C6	−159.2 (2)	C6—Fe1—C4—C3	−167.8 (2)
C4—Fe1—C10—C6	165.53 (12)	C1—Fe1—C4—C3	−81.43 (14)
C2—Fe1—C10—C6	46.2 (3)	C7—Fe1—C4—C3	46.7 (3)
C5—Fe1—C10—C6	123.08 (13)	C8—Fe1—C4—C3	77.89 (15)
C1—Fe1—C10—C6	81.19 (14)	C9—Fe1—C4—C5	−121.56 (13)
C7—Fe1—C10—C6	−37.52 (12)	C3—Fe1—C4—C5	118.72 (19)
C8—Fe1—C10—C6	−80.88 (13)	C10—Fe1—C4—C5	−79.78 (15)
C3—Fe1—C10—C9	−40.3 (3)	C2—Fe1—C4—C5	81.10 (14)
C4—Fe1—C10—C9	−75.49 (13)	C6—Fe1—C4—C5	−49.1 (3)
C2—Fe1—C10—C9	165.1 (2)	C1—Fe1—C4—C5	37.30 (13)
C5—Fe1—C10—C9	−117.94 (12)	C7—Fe1—C4—C5	165.4 (2)
C6—Fe1—C10—C9	118.98 (16)	C8—Fe1—C4—C5	−163.38 (13)
C1—Fe1—C10—C9	−159.83 (12)	C18—C17—O2—C20	−177.2 (2)
C7—Fe1—C10—C9	81.46 (12)	C16—C17—O2—C20	2.9 (3)

### Hydrogen-bond geometry (Å, °)

**Table:**

*D*—H···*A*	*D*—H	H···*A*	*D*···*A*	*D*—H···*A*
C13—H13···O1	0.93	2.50	2.830 (2)	101
C8—H8···O1^i^	0.93	2.60	3.345 (2)	137
C9—H9···O1^ii^	0.93	2.54	3.414 (2)	156
C12—H12···O1^ii^	0.93	2.46	3.346 (2)	160
C15—H15···O1^ii^	0.93	2.65	3.441 (2)	143
C19—H19···O2^iii^	0.93	2.59	3.472 (3)	157
C3—H3···Cg1^i^	0.93	3.24	3.808 (2)	121

Symmetry codes: (i) *x*+1/2, −*y*+1/2, −*z*+1; (ii) −*x*+1/2, *y*+1/2, *z*; (iii) −*x*, *y*−1/2, −*z*+1/2.
